# Steady-State-Preserving Simulation of Genetic Regulatory Systems

**DOI:** 10.1155/2017/2729683

**Published:** 2017-01-19

**Authors:** Ruqiang Zhang, Julius Osato Ehigie, Xilin Hou, Xiong You, Chunlu Yuan

**Affiliations:** ^1^College of Sciences, Nanjing Agricultural University, Nanjing 210095, China; ^2^College of Horticulture, Nanjing Agricultural University, Nanjing 210095, China; ^3^Department of Mathematics, University of Lagos, Lagos 23401, Nigeria

## Abstract

A novel family of exponential Runge-Kutta (expRK) methods are designed incorporating the stable steady-state structure of genetic regulatory systems. A natural and convenient approach to constructing new expRK methods on the base of traditional RK methods is provided. In the numerical integration of the one-gene, two-gene, and p53-mdm2 regulatory systems, the new expRK methods are shown to be more accurate than their prototype RK methods. Moreover, for nonstiff genetic regulatory systems, the expRK methods are more efficient than some traditional exponential RK integrators in the scientific literature.

## 1. Introduction

One of the challenges in systems biology is to understand how biochemical molecules, such as DNAs, mRNAs, and proteins, interact to form harmonic and uniform cellular systems which give rise to life (see [1, 2]). The synthetic genetic regulatory networks (GRNs) play an important role in the investigation of protein regulation processes in living organisms (see [3–6]). By introducing ordinary differential equations (ODEs) to describe the rates of change in the concentrations of biochemical molecules, such as mRNAs and proteins, more detailed understanding and insights of the dynamic behavior exhibited by biological systems can be achieved (see [7]). The first attempt to model the oscillation in genetic regulation in terms of ODEs was made by Goodwin [8]. A standard presentation of general regulatory dynamics can be found in the monographs by Thomas and D'Ari [9] and by Fall et al. [10]. Iwamoto et al. [11] presented a dynamical model of the DNA damage signaling pathway that includes p53 and whole cell cycle regulation and explored the relationship between p53 oscillation and cell fate selection. ODEs models admit mathematically qualitative and quantitative analysis to reveal the profound properties from steady states with stability, bistability, oscillation, and limit cycles to chaos (see [12–17] and the references therein).

A typical system of ODEs governing an *N*-gene activation-inhibition system has the form (see Polynikis et al. [15])

(1) Transcription:  r˙i=fiRp−γiri,Translation:  p˙i=fiPri−μipi,

where, for *i* = 1,…, *N*, *r*_*i*_ is the concentration of mRNA *R*_*i*_ produced by gene *g*_*i*_, *p*_*i*_ is the concentration of protein *P*_*i*_ translated from mRNA *R*_*i*_, *γ*_*i*_ is the degradation rate of *R*_*i*_, and *μ*_*i*_ is the degradation rate of *P*_*i*_. Function *f*_*i*_^*P*^(*r*_*i*_) is the translation function. Function *f*_*i*_^*R*^(*p*) is the regulation function, typically taking the form of a sum of products of functions *f*_*i*1_^*R*^(*p*_1_),…, *f*_*iN*_^*R*^(*p*_*N*_). If protein *P*_*j*_ has no effect on gene *g*_*i*_, *f*_*j*_^*R*^(*p*_*j*_) does not appear in *f*_*i*_^*R*^. The partial derivative ∂*f*_*i*_^*R*^/∂*p*_*j*_ > 0 if protein *P*_*j*_ is an* activator* of gene *g*_*i*_ and ∂*f*_*i*_^*R*^/∂*p*_*j*_ < 0 if protein *P*_*j*_ is an* inhibitor* of gene *g*_*i*_. The genetic regulatory system (1) can be written in matrix form

(2) r˙t=−Γrt+Fpt,p˙t=Krt−Mpt,

where *r*(*t*) = (*r*_1_(*t*),…, *r*_*N*_(*t*))^*T*^ and *p*(*t*) = (*p*_1_(*t*), *p*_2_(*t*),…, *p*_*N*_(*t*))^*T*^ are *N*-dimensional vectors representing the concentrations of mRNAs and proteins at time *t*, respectively, and *F*(*p*(*t*)) = (*F*_1_(*p*(*t*)),…, *F*_*N*_(*p*(*t*)))^*T*^, Γ = diag⁡(*γ*_1_,…, *γ*_*N*_), *M* = diag⁡(*μ*_1_,…, *μ*_*N*_), and *K* = diag⁡(*κ*_1_,…, *κ*_*N*_) are diagonal matrices.

The analytical solution of system (1) is in general not acquirable due to the nonlinearity of the functions *f*_*i*_^*P*^(*r*_*i*_) and *f*_*ij*_^*R*^(*p*_*j*_). Therefore, in order to explore the dynamics of the gene regulatory system (1), one usually resorts to numerical solution. For example, Shinto et al. [18] proposed a kinetic simulation model of metabolic pathways that describes the dynamic behaviors of metabolites in acetone-butanol-ethanol (ABE) production by* Clostridium saccharoperbutylacetonicum* N1-4 using a simulator WinBEST-KIT. So far, differential equations for genetic regulation are mostly solved by the classical four-stage Runge-Kutta (RK) method or by the Runge-Kutta-Fehlberg adaptive method (see Hairer et al. [19]). However, general-purpose RK methods have not taken into account the special structure of system (1) and fail to capture the dynamical features of the system effectively, especially in the long time simulation (see Figure 11). Thanks to new advances in the last two decades, new approaches have been developed aiming at preserving the intrinsic geometric or physical structures of the true solution. A comprehensive account of structure-preserving algorithms can be found in the monographs by Stuart and Humphries [20], Hairer et al. [21], and Wu et al. [22]. Recently Hochbruck and Ostermann [23] investigated exponential Runge-Kutta methods for initial value problems of parabolic differential equations. This type of methods simulates exactly the linear structure of the differential equations. Defterli et al. [24] and Weber et al. [25] considered discretizing and optimizing the so-called gene-environment networks based on usually finite data series.

From the dynamics point of view, there are two basic categories of genetic regulatory systems: Category 1 consists of systems having sustained oscillation, such as limit cycles; Category 2 consists of systems having steady states. For the genetic regulatory system (1) with a limit-cycle structure, You [26] proposed a new class of phase-fitted and amplification-fitted Runge-Kutta type methods which were shown to be more effective and more efficient than the traditional Runge-Kutta methods of the same order. Very recently, You et al. [27] developed a splitting approach for genetic regulatory systems with a stable steady state. In the numerical simulation, the new splitting methods constructed in that paper are shown to be remarkably more effective and more suitable for long-term computation with large steps than the general-purpose Runge-Kutta methods. In order to respect the oscillatory feature of the solution of some genetic regulatory systems, Chen et al. [28] developed a new type of exponentially fitted TDRK (EFTDRK) methods. Zhang et al. [29] constructed a family of phase-fitted symmetric splitting methods of order two and order four. The result of the numerical experiment on the Lotka–Volterra system shows that the new phase-fitted symmetric splitting methods are more effective than their prototype splitting methods and can preserve the invariant of the system in the long term compared with the classical Runge-Kutta method of order four.

The purpose of this paper is to develop a novel type of exponential RK methods for the simulation of genetic regulatory systems which have an asymptotically stable steady state. In Section 2 we present the general scheme of exponential Runge-Kutta (expRK) methods for solving initial value problems of ODEs based on a matrix form of the variation-of-constants formula. A convenient approach of transiting traditional RK methods into a special type of expRK methods is given. In Section 3 we integrate the above three regulatory systems by the new expRK methods as well as their prototype RK methods for comparison.  Section 4 is devoted to conclusive remarks. In Appendix, we analyze the linear stability and phase properties of the expRK methods.

## 2. Exponential Runge-Kutta Methods

### 2.1. Formulation of Exponential RK Methods for Systems with a Stable Steady-State Structure

Prior to dealing with the genetic regulatory system (2) numerically, we first consider the general initial value problem (IVP) of the autonomous system of ODEs

(3) y˙=fy,t>0,y0=y0,

where *y* : [0, +*∞*) → *ℝ* ^*d*^ and “$\dot{y}$” represents the derivative of *y* with respect to time. We make the following assumptions on system (3):

(**A1**) The origin *y* ^*∗*^ = 0 is a steady state of the system; that is, *f*(0) = 0.
(**A2**) The Jacobian (∂*f*/∂*y*)(0) has eigenvalues of negative real parts in a neighborhood of the origin. Then, according to the theorem in Section  8.5 of Hirsch et al. [30], the origin is an asymptotically stable steady state; that is, for every solution *y*(*t*) of system (3) through a point in the neighborhood of the steady state, lim_*t*→+*∞*_ *y*(*t*) = 0.
(**A3**) The function *f* : *ℝ* ^*d*^ → *ℝ* ^*d*^ is continuously differentiable and satisfies the Lipschitz condition; that is, there exists a constant *L* (called the* Lipschitz constant*) such that
  (4) $$\begin{array}{c} \left\|\;f\left(\;y\;\right)-f\left(\;z\;\right)\;\right\|\leq L\left\|\;y-z\;\right\| \end{array}$$
for all *y*, *z* ∈ *ℝ* ^*d*^.

Let us recall the general-purpose Runge-Kutta methods for IVP (3).

**Definition 1 .**

An *s*-stage Runge-Kutta (RK) method for system (3) has the scheme

(5) Yi=yn+h∑j=1saijfYj,i=1,…,s,yn+1=yn+h∑i=1sbifYi,

where *c*_*i*_, *a*_*ij*_, *b*_*i*_, *i*, *j* = 1,…, *s*, are real numbers.

Scheme (5) can be expressed briefly by the Butcher tableau of its coefficients

(6) $$\begin{array}{c} \begin{array}{cc} c & A\\ & b^{T} \end{array}=\begin{array}{cccc} c_{1} & a_{11} & \cdots & a_{1s}\\ \vdots & \vdots & \ddots & \vdots \\ c_{s} & a_{s1} & \cdots & a_{ss}\\ & b_{1} & \cdots & b_{s} \end{array}. \end{array}$$

The order conditions for the RK method (5) can be found in Hairer et al. [19]. Note that the general RK scheme (5) does not take into account the special structure of the equilibrium structure of the system so that the computational results are usually not satisfactory. In order to simulate more effectively system (3) with a steady state at the origin, we rewrite problem (3) in an equivalent form

(7) y˙−Ωy=gy,y0=y0,

where the matrix *Ω* = (∂*f*/∂*y*)(0), the function *g*(*y*) = *f*(*y*) − *Ωy* = *𝒪*(‖*y*‖^2^), ‖·‖ is the Euclidean norm, and *Ω* is called the* rate matrix* of system (3). The matrix form of the variation-of-constants formula for system (3) is given by

(8) ytn+μh=exp⁡μhΩytn+exp⁡μhΩ∫tntn+μhexp⁡−ξ−tnΩgyξdξ,

where *μ* and *h* are real numbers and *t*_*n*_ = *nh*, *n* = 0,1,…. Approximating the integral on the right-hand side of (8) by some effective quadrature formulas leads to a numerical integrator. In general we have the following definition of the so-called exponential Runge-Kutta methods.

**Definition 2 .**

An *s*-stage exponential Runge-Kutta method for system (3) has the scheme

(9) Yi=exp⁡ciVyn+∑j=1saijVhfYj−VYj,i=1,…,s,yn+1=exp⁡Vyn+∑i=1sbiVhfYi−VYi,

where *c*_*i*_, *i* = 1,…, *s*, are real numbers and *a*_*ij*_(*V*) and *b*_*i*_(*V*), *i*, *j* = 1,…, *s*, are real *d* × *d* matrix-valued functions of matrix *V* = *hΩ*.

It is convenient to express scheme (9) by the Butcher tableau

(10) cexp⁡cVAVexp⁡VbTV=c1expc1Va11V⋯a11V⋮⋮⋮⋱⋮csexpc1Vas1V⋯assVexp⁡Vb1V⋯bsV,

where exp⁡(*cV*) = (exp⁡(*c*_1_ *V*)^*T*^,…, exp⁡(*c*_*s*_ *V*)^*T*^)^*T*^. For a comprehensive review of exponential integrators with the construction, analysis of convergence and error bounds, order conditions, example integrators, and applications, the reader is referred to Hochbruck and Ostermann [23].

It is noted that if we need to integrate a system *z*′ = *ψ*(*z*) near a stable steady state *z* ^*∗*^ ≠ 0, the exponential RK scheme (9) should take the form

(11) yn=zn−z∗,Yi=exp⁡ciVyn+∑j=1saijVhψYj+z∗−VYj,i=1,…,s,yn+1=exp⁡Vyn+∑i=1sbiVhψYi+z∗−VYi,zn+1=yn+1+z∗,

where *V* = *hΩ* with *Ω* = (∂*ψ*/∂*z*)(*z* ^*∗*^).

### 2.2. A Special Class of Exponential RK Methods

Based on an RK method (5), we can formulate, as a special case of the exponential RK method (9), the following scheme for system (3):

(12) Yi=exp⁡ciVyn+∑j=1saijexp⁡ci−cjVhfYj−VYj,i=1,…,s,yn+1=exp⁡Vyn+∑i=1sbiexp⁡1−ciVhfYi−VYi,

where *c*_*i*_, *a*_*ij*_, and *b*_*i*_, *i*, *j* = 1,…, *s*, are the constant coefficients of the RK method (5). Note that as *Ω* → 0 (*V* → 0), scheme (12) reduces to the RK method (5). The latter is called the* prototype RK method* of the former. In the sequel of this paper, scheme (12) will be referred to as an* expRK method*. Its Butcher tableau can be written as follows:
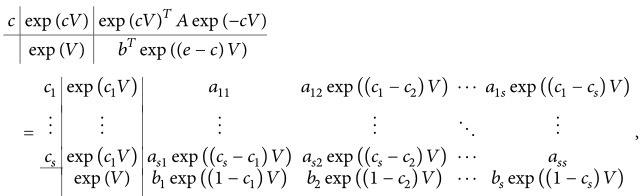
(13)where *e* = (1,…, 1)^*T*^, exp⁡((*e* − *c*)*V* = (exp⁡((1 − *c*_1_)*V*)^*T*^,…, exp⁡((1 − *c*_*s*_)*V*)^*T*^)^*T*^, and exp⁡(*cV*)^*T*^ *A*exp⁡(−*cV*) = (exp⁡((*c*_*i*_ − *c*_*j*_)*V*)*a*_*ij*_)_*s*×*s*_.

In Kronecker's notation, scheme (12) can be written as

(14) Y=exp⁡cVyn⊗e+exp⁡cVA⊗Idexp⁡−cVhfY−VY,yn+1=exp⁡Vyn+exp⁡VbT⊗Idexp⁡−cVhfY−VY,

where *I*_*d*_ is the *d* × *d* unit matrix and exp⁡(−*cV*)(*hf*(*Y*) − *VY*) = (exp⁡((−*c*_1_)*V*)^*T*^(*hf*(*Y*_1_) − *VY*_1_),…, exp⁡((−*c*_*s*_)*V*)^*T*^(*hf*(*Y*_*s*_) − *VY*_*s*_))^*T*^.

Among simple examples are the following:

(a) The* exponential Euler method* (explicit), denoted by expEuler:
  (15) $$\begin{array}{c} y_{n+1}=\exp\left(\;V\;\right)y_{n}+\exp\left(\;V\;\right)\left(\;hf\left(\;y_{n}\;\right)-Vy_{n}\;\right) \end{array}$$
(b) The* exponential backward Euler method* (implicit), denoted by expImEuler:
  (16) $$\begin{array}{c} y_{n+1}=\exp\left(\;V\;\right)y_{n}+\left(\;hf\left(\;y_{n+1}\;\right)-Vy_{n+1}\;\right) \end{array}$$
(c) The* exponential trapezoidal rule* (implicit), denoted by expTrap:
  (17) yn+1=expVyn+expV2hfyn−Vyn+12hfyn+1−Vyn+1
(d) The* exponential Heun rule* (explicit), denoted by expHeun:
  (18) yp=exp⁡Vyn+exp⁡Vhfyn−Vyn,yc=exp⁡Vyn+hfyp−Vyp,yn+1=yp+yc2
(e) The* exponential midpoint rule* (implicit), denoted by expMid:
  (19) yn+1=exp⁡Vyn+exp⁡V2·hfexp⁡V/2yn+exp⁡−V/2yn+12−12VexpV2yn+exp−V2yn+1

Two typical expRK methods, denoted by expRK3/8 and expRK4, have the prototype RK methods of order four, denoted by RK3/8 and RK4, respectively, whose respective Butcher tableaux are given in Page 138 of [19]

(20) $$\begin{array}{c} \begin{array}{ccccc} 0 & & & & \\ \frac{1}{3} & \frac{1}{3} & & & \\ \frac{2}{3} & -\frac{1}{3} & 1 & & \\ 1 & 1 & -1 & 1 & \\ & \frac{1}{8} & \frac{3}{8} & \frac{3}{8} & \frac{1}{8} \end{array}, \end{array}$$

(21) $$\begin{array}{c} \begin{array}{ccccc} 0 & & & & \\ \frac{1}{2} & \frac{1}{2} & & & \\ \frac{1}{2} & 0 & \frac{1}{2} & & \\ 1 & 0 & 0 & 1 & \\ & \frac{1}{6} & \frac{2}{6} & \frac{2}{6} & \frac{1}{6} \end{array} \end{array}$$

The (algebraic) order is a measure of the accuracy of numerical method. A method is said to have* order p* if its local error LE = *𝒪*(*h* ^*p*+1^).

**Theorem 3 .**

The expRK method (12) has the same algebraic order as its prototype RK method.

**Proof**

The conclusion follows by expanding the exponential functions exp⁡(*c*_*i*_ *V*), exp⁡((*c*_*i*_ − *c*_*j*_)*V*), and exp⁡((1 − *c*_*i*_)*V*) in scheme (12) in series of *V* = *hΩ*, hence in series of *h*, and comparing this series with that of the true solution.

The next theorem asserts that exponential RK method (12) preserves the steady state of system (3).

**Theorem 4 .**

Suppose that *f* in system (3) satisfies the Lipschitz condition and the origin is a steady state of the system; that is, *f*(0) = 0. Then the origin is also a fixed point of the expRK method (12) for small step size *h*.

The proof is given in Appendix A.

In order to apply the expRK method (9) or (12) to system (2), we first use a coordinate transform *u*(*t*) = *r*(*t*) − *r* ^*∗*^, *v*(*t*) = *p*(*t*) − *p* ^*∗*^ to translate the steady state (*r* ^*∗*^, *p* ^*∗*^) of the system to the origin and yields

(22) u˙t=−Γut+F′p∗vt+Gvt,v˙t=Kut−Mvt,

where *F*′(*p* ^*∗*^) is the Jacobian matrix of *F*(*p*) at point *p* ^*∗*^ and *G*(*v*(*t*)) = *F*(*p* ^*∗*^ + *v*(*t*)) − *F*′(*p* ^*∗*^)*v*(*t*) − *F*(*p* ^*∗*^). Then system (2) can be written in the form (7) with *y*(*t*) = (*u*(*t*), *v*(*t*))^*T*^ where the rate matrix

(23) $$\begin{array}{c} \Omega =\left(\begin{array}{cc} -\Gamma & F^{\prime }\left(p^{*}\right)\\ K & -M \end{array}\right) \end{array}$$

and the function *g*(*y*) = (*G*(*v*)^*T*^, 0)^*T*^.

## 3. Numerical Illustrations

From Theorem 4, contrast to traditional RK methods, exponential RK methods, especially expRK methods, retain the rate of growth, phase, and amplification of the exact solution of the test equation (B.1) without error. Then it is reasonable to expect expRK (12) to be more effective than their prototype RK methods. In this section, in order to compare their effectiveness, we apply them to three test systems—one-gene self-regulation system, two-gene cross-regulation system, and the p53-mdm2 system.

*(I) A One-Gene System of Self-Regulation*. The first model we consider is the one-gene system with self-regulation given by

(24) r˙t=−γrt+Fpt,p˙t=−μpt+κrt,

where *F*(*p*(*t*)) = *α*/(1 + *p*(*t*)^2^/*θ* ^2^) represents the action of an inhibitory protein that acts as a dimer and *γ*, *μ*, *κ*, *α*, *θ* are positive constants. For a similar model with delays see Xiao and Cao [31]. 

*(II) A Two-Gene System with Cross-Regulation*. The second model is a two-gene activation-inhibition system with cross-regulation (studied by Polynikis et al. [15], Widder et al. [17], Chen et al. [32], You [26], and You et al. [27])

(25) r˙1=m1H+p2;θ2,n2−γ1r1,r˙2=m2H−p1;θ1,n1−γ2r2,p˙1=κ1r1−μ1p1,p˙2=κ2r2−μ2p2,

where, for *i* = 1,2, *r*_*i*_ is the concentration of mRNA *R*_*i*_ produced by gene *g*_*i*_, *p*_*i*_ is the concentration of protein *P*_*i*_, *m*_*i*_ is the maximal transcription rate of gene *g*_*i*_, *κ*_*i*_ is the translation rate of mRNA *R*_*i*_, and *γ*_*i*_ and *μ*_*i*_ are the degradation rates of mRNA *R*_*i*_ and protein *P*_*i*_, respectively. The functions

(26) H+p2;θ2,n2=p2n2θ2n2+p2n2,H−p1;θ1,n1=θ1n1θ1n1+p1n1

are the* Hill functions* of activation and repression, respectively. The parameters *θ*_1_, *θ*_2_ are the* expression thresholds*. The integer value of *n*_*i*_  (*i* = 1,2), called the* Hill coefficient*, stands for the number of protein monomers required for saturation of binding to DNA. It is easy to see that the activation function *H* ^+^ is increasing in *p*_2_ and the repression function *H* ^−^ is decreasing in *p*_1_. 

*(III) The p53-mdm2 System*. The third model is for the damped oscillation of the p53-mdm2 regulatory pathway. Strictly speaking, this system is not of the form (1). We adopt this model since its solutions also have a stable steady-state structure of interest. The system, given by van Leeuwen et al. [33] with the small transient stress stimulus *S*(*t*) = 0, has the form

(27) P˙I=sp+jaPA−dpPI−kcPIM+jcC,M˙=sm0+sm1PI+sm2PAPI+PA+Km+ku+jcC−dm+kcPIM,C˙=kcPIM−jc+kuC,P˙A=−ja+dpPA,

where *P*_*I*_ represents the concentration of the p53 tumor suppressor, *M* (mdm2) is the concentration of the p53's main negative regulator, *C* is the concentration of the p53-mdm2 complex, *P*_*A*_ is the concentration of an active form of p53 that is resistant against mdm2-mediated degradation, *s*_*∗*_  (*∗* = *p*, *m*0, *m*1) are de novo synthesis rates, *k*_*∗*_  (*∗* = *a*, *c*, *u*) are production rates, *j*_*∗*_  (*∗* = *a*, *c*) are reverse reactions (e.g., dephosphorylation), *d*_*p*_ is the degradation rate of active p53, and *K*_*m*_ is the saturation coefficient.

### 3.1. Accuracy Test

#### 3.1.1. The One-Gene System

Steady states of system (24) can be determined by the cubic equation (1/*θ* ^2^)*p* ^*∗*^^3^ + *p* ^*∗*^ + *ακ*/*γμ* = 0 and the relation *r* ^*∗*^ = (*μ*/*κ*)*p* ^*∗*^. If the system is written in the form (7), the rate matrix

(28) $$\begin{array}{c} \Omega =\left(\begin{array}{cc} -\gamma & -\frac{2\alpha p^{*}/\theta^{2}}{{\left(1+{p^{*}}^{2}/\theta^{2}\right)}^{2}}\\ \kappa & -\mu \end{array}\right), \end{array}$$

and the function

(29) $$\begin{array}{c} g\left(\;y\;\right)=\left(\begin{array}{c} \frac{\alpha }{1+{\left(y_{2}+p^{*}\right)}^{2}/\theta^{2}}+\frac{2\alpha p^{*}/\theta^{2}}{{\left(1+{p^{*}}^{2}/\theta^{2}\right)}^{2}}y_{2}\\ 0 \end{array}\right), \end{array}$$

where *y* = (*y*_1_, *y*_2_)^*T*^ = (*r* − *r* ^*∗*^, *p* − *p* ^*∗*^). With the parameter values (provided by [31])

**Figure 1:**
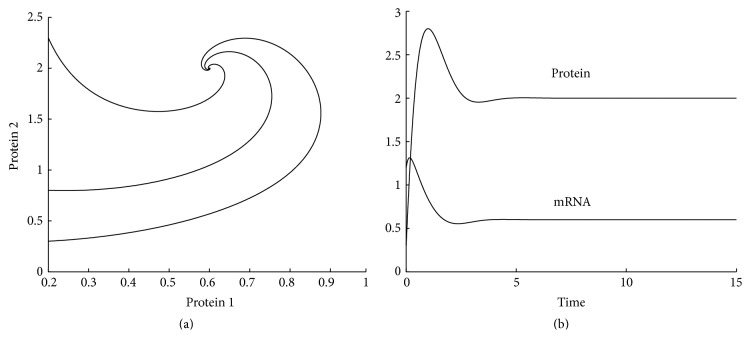
One-gene system: (a) solution trajectories; (b) time evolution of concentrations of mRNA and protein.

(30) α=3,γ=1,μ=1.5,κ=5,θ=1,

this system has a unique positive steady state (*r* ^*∗*^, *p* ^*∗*^) = (0.6,2) where the rate matrix *Ω* has eigenvalues *λ*_1,2_ = −1.2500 ± 2.9767*i*, where *i* is the imaginary unit satisfying *i* ^2^ = −1. Since the two eigenvalues both have negative real parts, the steady state is asymptotically stable.  Figure 1(a) shows three solution trajectories on the phase plane starting at (*r*(0), *p*(0)) = (0.2,0.3), (0.2,0.8), (0.2,2.3), respectively.  Figure 1(b) shows the time evolution of concentrations of mRNA and protein starting at (*r*(0), *p*(0)) = (1.2,0.3).

**Figure 2:**
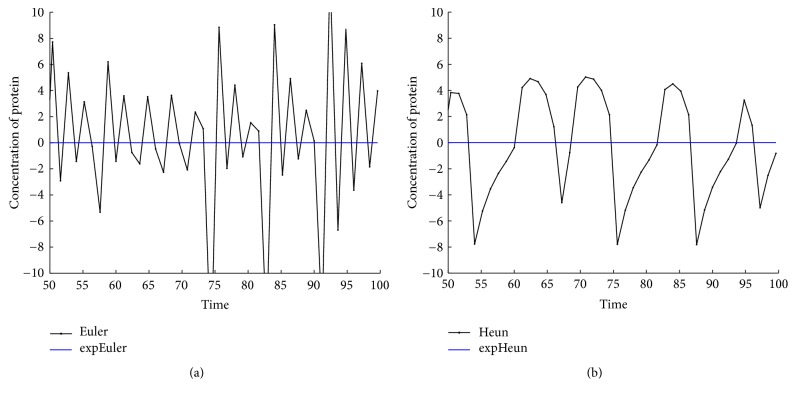
Accuracy comparison for the one-gene system: (a) Euler and expEuler; (b) Heun and expHeun with step size *h* = 1.2.

**Figure 3:**
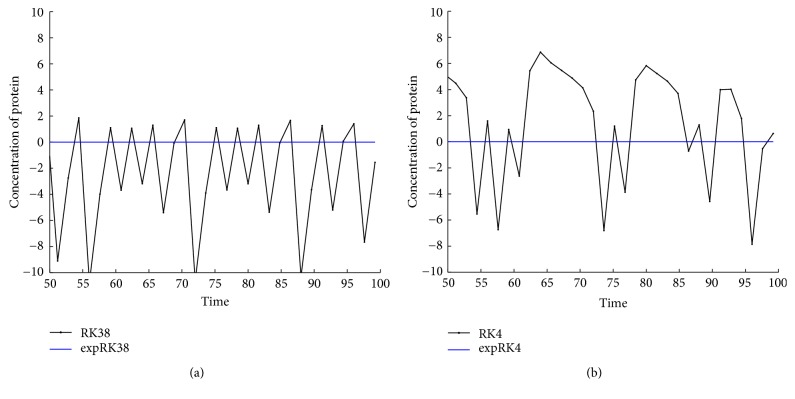
Accuracy comparison for the one-gene system: (a) RK3/8 and expRK3/8; (b) RK4 and expRK4 with step size *h* = 1.6.

With the above values of parameters and initial data, we integrate system (25) on the time interval [0,100] by the methods expEuler, expHeun, expRK3/8, and expRK4 as well as their corresponding prototype methods. We plot the error growth of the protein on the time interval [50,100]. The numerical results are presented in Figures 2 and 3.

The system is solved on the time interval [0,100] with initial values of mRNA and protein *r*(0) = 0.6 and *p*(0) = 0.8 and with different step sizes. The numerical results are presented in Tables 1 and 2.

#### 3.1.2. The Two-Gene System

The steady states *y* ^*∗*^ = (*r*_1_^*∗*^, *r*_2_^*∗*^, *p*_1_^*∗*^, *p*_2_^*∗*^)^*T*^ of system (25) are determined by the equations

(31) θ2n2p1∗p1∗n1+θ1n1n2+p1∗−m1k1γ1μ1m2k2γ2μ2θ1n1n2=0,

(32) r1∗=μ1κ1p1∗,p2∗=m2κ2θ1n1γ2μ2θ1n1+p1∗n1.

Putting in the form (7), system (25) has the rate matrix

(33) Ω=∂f∂yy∗=−γ100m1n2θ2n2p2∗n2−1θ2n2+p2∗n220−γ2−m2n1θ1n1p1∗n1−1θ1n1+p1∗n120κ10−μ100κ20−μ2

and the function

(34) gy=m1p2n2θ2n2+y4+p2∗n2−γ1r1∗−m1n2θ2n2p2∗n2−1θ2n2+p2∗n22y4m2θ1n1θ1n1+y3+p1∗n1−γ2r2∗−m2n1θ1n1p1∗n1−1θ1n1+p1∗n12y300

with *y* = (*y*_1_, *y*_2_, *y*_3_, *y*_4_)^*T*^ = (*r*_1_ − *r*_1_^*∗*^, *r*_2_ − *r*_2_^*∗*^, *p*_1_ − *p*_1_^*∗*^, *p*_4_ − *p*_4_^*∗*^)^*T*^. The characteristic equation of the rate matrix *Ω* is

(35) $$\begin{array}{c} \left(\;\lambda +\gamma_{1}\;\right)\left(\;\lambda +\gamma_{2}\;\right)\left(\;\lambda +\mu_{1}\;\right)\left(\;\lambda +\mu_{2}\;\right)+D=0, \end{array}$$

where

(36) $$\begin{array}{c} D=m_{1}m_{2}\kappa_{1}\kappa_{2}\frac{n_{1}n_{2}{p_{1}^{*}}^{n_{1}-1}{p_{2}^{*}}^{n_{2}-1}\theta_{1}^{n_{1}}\theta_{2}^{n_{2}}}{{\left({p_{1}^{*}}^{n_{1}}+\theta_{1}^{n_{1}}\right)}^{2}{\left({p_{2}^{*}}^{n_{2}}+\theta_{2}^{n_{2}}\right)}^{2}}. \end{array}$$

For a certain value of *D* = *D*_Hopf_, the real part of one of the eigenvalues crosses zero, indicating a loss of stability through a Hopf bifurcation. For *n*_1_ > 1 and *n*_2_ > 1, [17] has calculated this value explicitly as

(37) DHopf=γ1+γ2γ1+μ1γ2+μ1γ1+μ2γ2+μ2μ1+μ2γ1+γ2+μ1+μ22.

In our experiment, we take the values of parameters as follows:

(38) n1=n2=3,m1=m2=1.8,k1=k2=1,γ1=γ2=1,μ1=μ2=1,θ1=θ2=0.6542.

**Figure 4:**
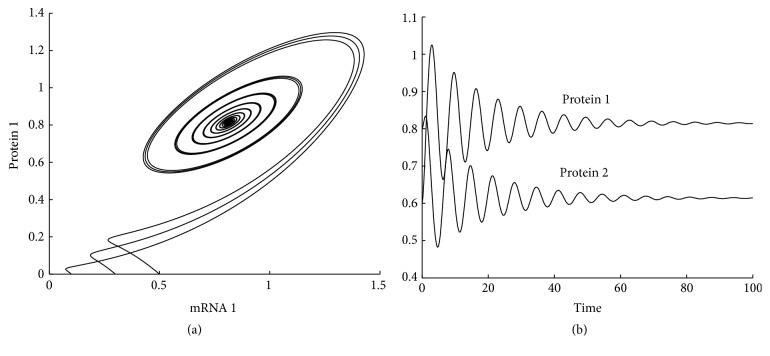
Two-gene system: (a) phase trajectories projected on mRNA 1-protein 1 plane; (b) time evolution of concentrations of proteins.

Figure 4(a) shows three solution trajectories projected on the mRNA 1-protein 1 plane starting at (*r*_1_(0), *r*_2_(0), *p*_1_(0), *p*_2_(0)) = (0.1,0.5,0, 0), (0.3,0.3,0, 0), and (0.5,0.2,0, 0), respectively.  Figure 4(b) shows the time evolution of concentrations of protein 1 and protein 2.

We solve (31) for *p*_1_^*∗*^ by Newton's iteration and then substitute it into (32) obtaining

(39) r1∗=0.81471271066221,r2∗=0.61403210214378,p1∗=0.81471271066221,p2∗=0.61403210214378.

The rate matrix (33) has the eigenvalues with negative real parts:

(40) λ1,2=−1.94911366876016±0.94911366876016i,λ3,4=−0.05088633123984±0.94911366876016i.

Therefore the steady state *y* ^*∗*^ is asymptotically stable.

**Figure 5:**
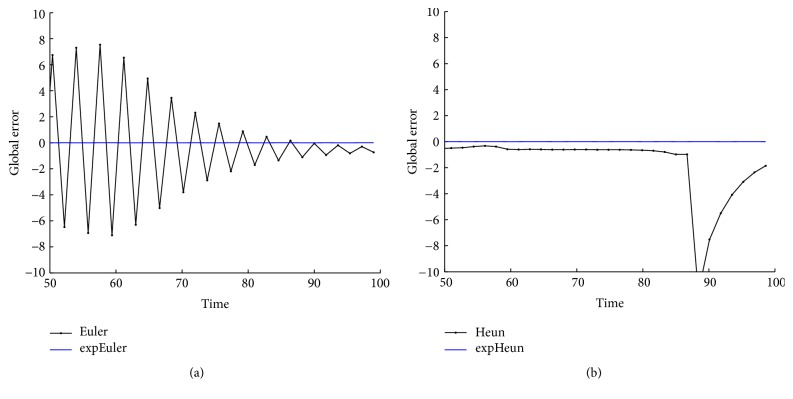
Accuracy comparison for the two-gene system: (a) Euler and expEuler; (b) Heun and expHeun.

**Figure 6:**
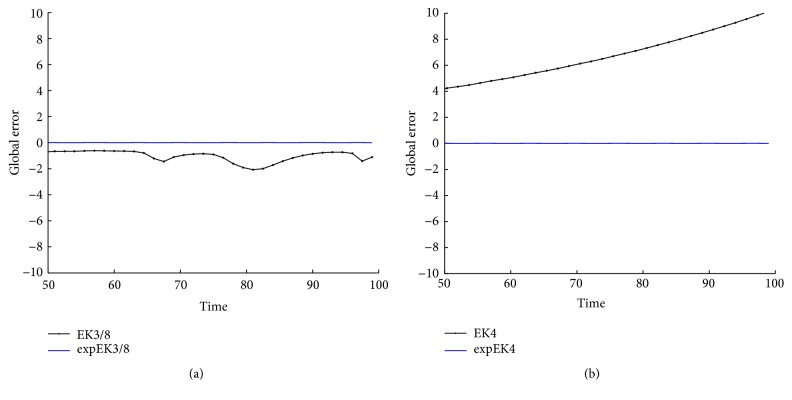
Accuracy comparison for the two-gene system: (a) RK3/8 and expRK3/8; (b) RK4 and expRK4.

With the values of parameters (38) and initial data and initial values *r*_1_(0) = 0.6, *r*_2_(0) = 0.8, *p*_1_(0) = 0, and *p*_2_(0) = 0, we integrate system (25) on the time interval [0,100] by the methods expEuler, expHeun, expRK3/8, and expRK4 as well as their corresponding prototype methods with the step sizes *h* = 1.8, *h* = 1.7, *h* = 1.5, *h* = 1.678, respectively. In Figures 5 and 6, we plot the global error growth of protein 1 on the time interval [50,100].

In Tables 3 and 4, average errors are compared for differential step sizes.

#### 3.1.3. The p53-mdm2 System

The steady state *y* ^*∗*^ = (*P*_*I*_^*∗*^, *M* ^*∗*^, *C* ^*∗*^, *P*_*A*_^*∗*^) is determined by the following equations:

(41) sp−dpPI∗−kckudmjc+kuPI∗sm0+sm1PI∗+KmPI∗=0,M∗=sm0dm+sm1PI∗dmPI∗+Km,C∗=kcjc+kuPI∗M∗,PA∗=0.

Putting in the form (7), system (27) has the rate matrix
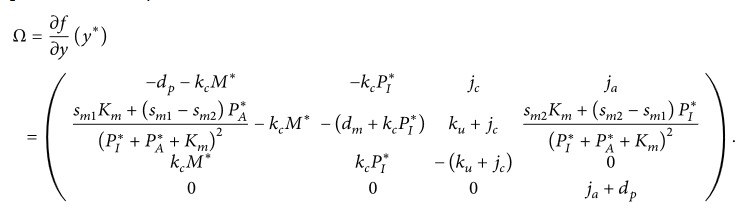
(42)and the function

(43) $$\begin{array}{c} g\left(\;y\;\right)=\left(\begin{array}{c} -k_{c}y_{1}y_{2}\\ g_{2}\left(y\right)\\ k_{c}y_{1}y_{2}\\ 0 \end{array}\right), \end{array}$$

where *y* = (*y*_1_, *y*_2_, *y*_3_, *y*_4_)^*T*^ = (*P*_*I*_ − *P*_*I*_^*∗*^, *M* − *M* ^*∗*^, *C* − *C* ^*∗*^, *P*_*A*_ − *P*_*A*_^*∗*^)^*T*^, and

(44) $$\begin{array}{c} g_{2}\left(\;y\;\right)=-\frac{\left(\left(\left(s_{m1}-s_{m2}\right)P_{A}^{*}+s_{m1}K_{m}\right)y_{1}+\left(\left(s_{m2}-s_{m1}\right)P_{I}^{*}+s_{m2}K_{m}\right)y_{4}\right)\left(y_{1}+y_{4}\right)}{\left(y_{1}+P_{I}^{*}+y_{4}+P_{A}^{*}+K_{m}\right){\left(P_{I}^{*}+P_{A}^{*}+K_{m}\right)}^{2}}-k_{c}y_{1}y_{2}. \end{array}$$

We use the parameter values (see [33]) as follows:

(45) sm0=2×10−3 nM min−1,ka=20 min−1,ja=0.2 min−1,sm1=0.15 nM min−1,kc=4 min−1 nM−1,jc=2×10−3 min−1,sm2=0.2 nM min−1,ku=0.4 min−1,dm=0.4 min−1,sp=1.4 nM min−1,Km=100 nM,dp=2×10−4 min−1.

For simplicity, we take the small function *S*(*t*) ≡ 0. The system has a unique steady state (*P*_*I*_^*∗*^, *M* ^*∗*^, *C* ^*∗*^, *P*_*A*_^*∗*^) = (9.42094,0.0372868,3.49529,0). The rate matrix *Ω* has the eigenvalues

(46) λ1=−38.4766,λ2,3=−0.0028±0.0220i,λ4=−0.2002.

Since all the eigenvalues have negative real parts, the steady state is asymptotically stable.

**Figure 7:**
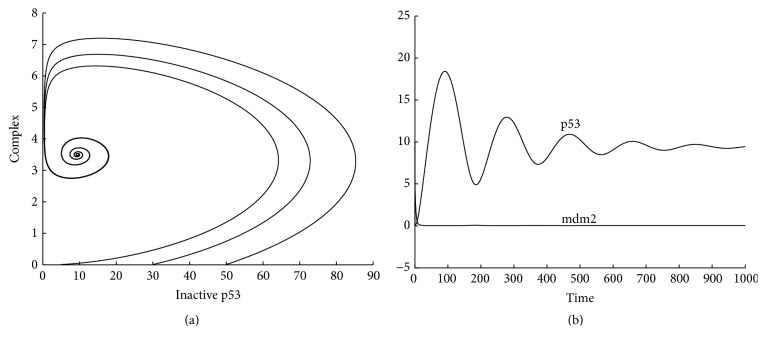
p53-mdm2 system: (a) phase curves projected on the inactive p53-complex plane; (b) time evolution of concentrations of p53 and mdm2.

Figure 7(a) shows three solution trajectories projected on the inactive p53-complex plane starting at (*P*_*I*_(0), *M*_0_(0), *C*_0_(0), *P*_*A*_(0)) = (5,0.01,0, 0), (30,0.01,0, 0), (15,0.01,0, 0), respectively.  Figure 7(b) shows the time evolution of concentrations of p53 and mdm2.

**Figure 8:**
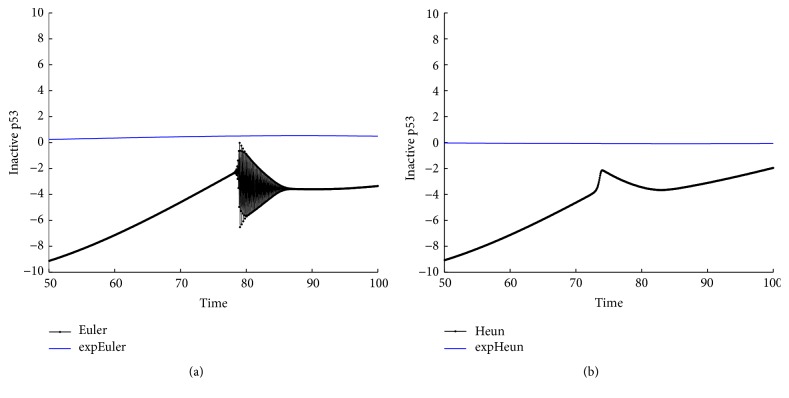
Accuracy comparison for the p53-mdm2 system: (a) Euler and expEuler; (b) Heun and expHeun.

**Figure 9:**
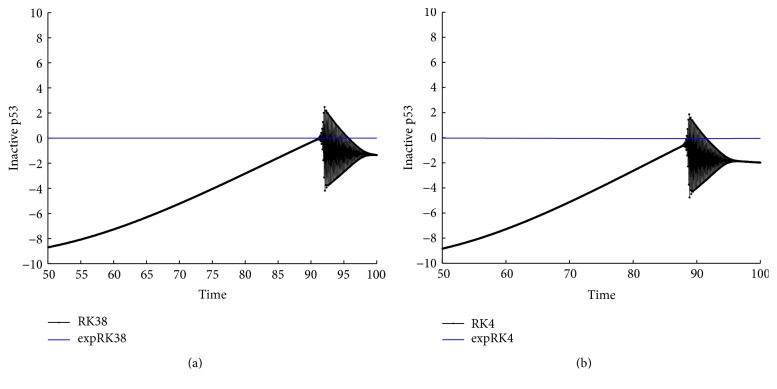
Accuracy comparison for the p53-mdm2 system: (a) RK3/8 and expRK3/8; (b) RK4 and expRK4.

With the values of parameters in (45) and initial data and initial values *P*_*I*_(0) = 10, *M*(0) = 0.1, *C*(0) = 3, and *P*_*A*_(0) = 0,8, we integrate system (25) on the time interval [0,100] by the methods expEuler, expHeun, expRK3/8, and expRK4 as well as their corresponding prototype methods with the step size *h* = 1/16. In Figures 8 and 9, we plot the global error growth of the inactive p53 on the time interval [50,100].

The problem is solved on the interval [0,50] for differential step sizes and the average errors are presented in Tables 5 and 6.

From Figures 2, 3, 5, 6, 8, and 9 and Tables 1 [Table tab2] [Table tab3] [Table tab4] [Table tab5]–6, we can see that the new expRK methods expEuler, expHeun, expRK3/8, and expRK4 are much more accurate than their corresponding prototype methods.

### 3.2. Efficiency Test

In this subsection we will compare the simulation efficiency of the newly constructed exponential RK methods with some famous exponential integrators. The integrators we choose for comparison are listed as follows:

- expRK3/8: the expRK method defined by (12) whose prototype RK method is given by (20)
- expRK4: the expRK method defined by (12) whose prototype RK method is given by (21)
- COX-MATTHEWS: the exponential RK method given by Cox and Matthews [34]
- KROGSTAD: the exponential RK method given by Krogstad [35]
- STREHMEL-WEINER: the exponential RK method given by Strehmel [36] (Example  4.5.5)
- HOCHBRUCK-OSTERMANN: the exponential RK method given by Hochbruck and Ostermann [23]

**Figure 10:**
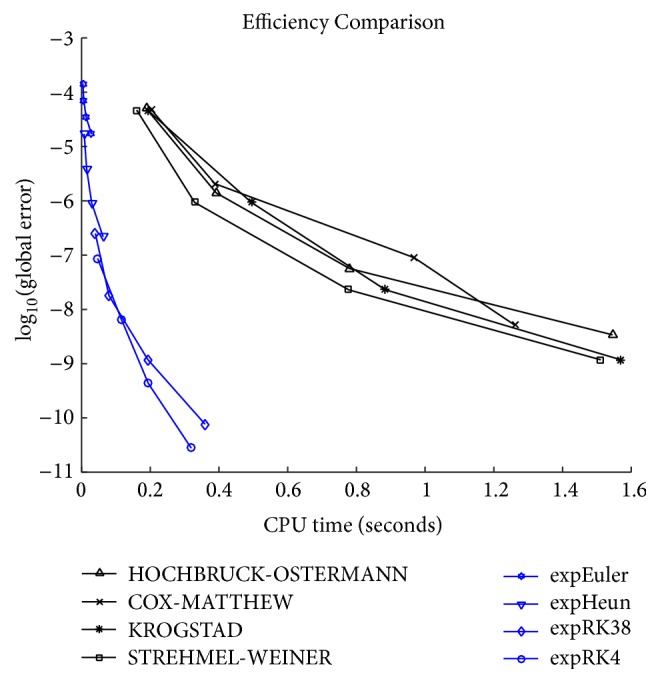
Efficiency curves with step sizes *h* = 1/2^*j*^, *j* = 1,2, 3,4.

The criterion for the efficiency is the digital logarithm of the global error against the CPU-time consumed. System (25) with parameters (38) is solved on the time interval [0,100] with the step sizes *h* = 1/2^*j*^, *j* = 1,2, 3,4. The numerical results are displayed in Figure 10, where we can see that the new exponential RK methods expEuler, expHeun, expRK3/8, and expRK4 are much more efficient than the other exponential RK methods we select from the literature. For the two-gene system, among all the exponent integrators we consider, expEuler, though the simplest, turns out to be the most efficient. It is also interesting to observe that as integration step size decreases from *h* = 1/2, expRK4 cannot produce smaller error, just increasing the computation effort.

## 4. Conclusions and Discussions

**Figure 11:**
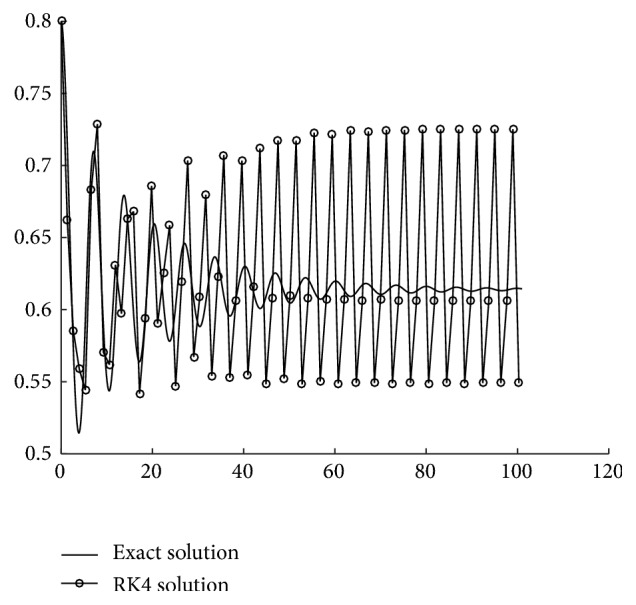
RK4 simulation with step size *h* = 1.32 compared with the exact solution.

Most genetic regulatory systems carry their own structures, such as stable steady states and sustained oscillation, bistability. Traditional Runge-Kutta (RK) methods have not taken into account these characteristic structures and may give misleading information. To see this, one only needs to integrate the two-gene system (25) by RK4 with step size *h* = 1.32. The simulation result, as presented in Figure 11, is qualitatively wrong. The exponential RK methods for system (3) originate from the discretization of the matrix form of variation-of-constants formula (8). This type of integrators has the property that they can integrate exactly linear systems of ODEs and thus can preserve the steady-state structure of system (3). From the numerical results presented in Section 3, an expRK method is more accurate than its traditional prototype RK method for long-term simulation with large step sizes. On the other hand, despite the simple form, the new expRK methods considered in this paper are tested to be more efficient than those prominent exponential RK methods when they are applied to genetic regulatory systems. The following advantages contribute to the high accuracy and high efficiency of the expRK methods compared to the classical RK methods.

(a) The scheme of the expRK methods recovers by the exponential functions the principal oscillatory structure of the true solution, which is contained in linear part (the Jacobian) of the system.

(b) The construction of the scheme is very simple and the coefficients are immediately obtained from a classical Runge-Kutta (RK) method.

(c) As shown in Appendix, the expRK methods (RK3/8 and RK4) have distortion and dissipation of the same order as their prototype RK methods but have dispersion of one order higher than their prototype RK methods. As the principal frequency is estimated accurately enough, that is, the ratio of the error of estimation and the testing frequency |*r* | ≪ 1, the coefficients of the leading terms of distortion and dissipation of the new expRK methods are much less than those of their prototype RK methods. Moreover, if *ω* is known to be the exact frequency (*r* = 1), expRK methods become zero-distortive, zero-dispersive, and zero-dissipative.

Although there may exist other methods of higher algebraic order that have higher accuracy, the expRK methods (12) are the most natural ones and are the most convenient to use. Note also that, similar to the approach of ode45, we can control the step by embedding two expRK methods of orders *p* and *p* + 1 into a pair to achieve higher efficiency.

Indeed, the test problem (25) is assumed to be nonstiff and this may be why our expRK methods outperform the existing exponential Runge-Kutta methods of Cox and Matthews, Hochbruck and Ostermann, Krogstad, and Stehmel and Weiner. The consideration of expRK methods for stiff genetic regulatory system (such as the p53-mdm2 systems, whose Jacobian possesses eigenvalues with large negative real parts or with purely imaginary eigenvalues of large modulus) is an interesting theme for future work. These systems contain different time scales due to coexistence of fast reactions and slow reactions which are frequently encountered in real cell processes. The expRK methods adapted to stiff systems will have a more delicate scheme to incorporate the stiff structure.

## Figures and Tables

**Figure 12:**
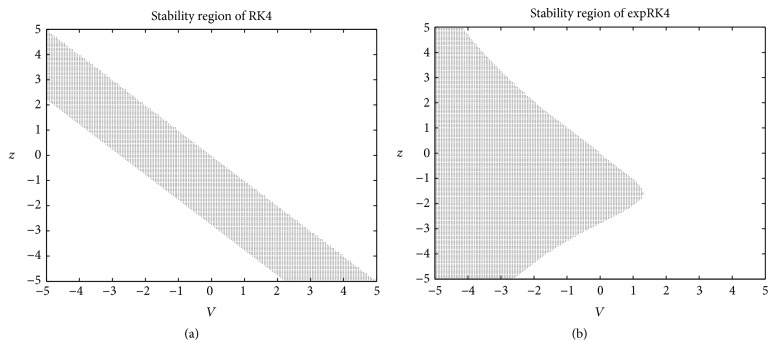
(a) Stability region of RK4; (b) stability region of expRK4.

**Table 1:** One-gene system: comparison of average errors for Euler, expEuler, Heun, and expEuler methods.

Step size	Euler	expEuler	Heun	expHeun
1/4	3.5849 × 10^−2^	1.1696 × 10^−3^	2.1384 × 10^−2^	1.5011 × 10^−4^
1/8	2.6231 × 10^−2^	5.2015 × 10^−4^	2.0930 × 10^−2^	5.7342 × 10^−5^
1/16	2.2715 × 10^−2^	2.3383 × 10^−4^	2.0539 × 10^−2^	3.8767 × 10^−5^
1/32	2.1259 × 10^−2^	9.8536 × 10^−5^	2.0284 × 10^−2^	3.5349 × 10^−5^

**Table 2 tab2:** One-gene system: comparison of average errors for RK3/8, expRK3/8, RK4, and expRK4 methods.

Step size	RK3/8	expRK3/8	RK4	expRK4
1/4	3.0743 × 10^−2^	3.1270 × 10^−5^	3.1713 × 10^−2^	2.2006 × 10^−4^
1/8	2.5309 × 10^−2^	3.4131 × 10^−5^	2.5490 × 10^−2^	3.4655 × 10^−5^
1/16	2.2533 × 10^−2^	3.4373 × 10^−5^	2.2569 × 10^−2^	3.4403 × 10^−5^
1/32	2.1219 × 10^−2^	3.4407 × 10^−5^	2.1227 × 10^−2^	3.4408 × 10^−5^

**Table 3 tab3:** Two-gene system: comparison of average errors for Euler, expEuler, Heun, and expHeun methods.

Step size	Euler	expEuler	Heun	expHeun
1	1.6832	4.4456 × 10^−3^	2.9451 × 10^−1^	2.1871 × 10^−3^
1/2	9.21627 × 10^−1^	2.4275 × 10^−3^	4.0230 × 10^−2^	1.0848 × 10^−3^
1/4	5.1922 × 10^−1^	1.4028 × 10^−3^	1.0096 × 10^−2^	9.5000 × 10^−4^
1/8	1.8775 × 10^−1^	1.0103 × 10^−3^	2.7588 × 10^−3^	9.4565 × 10^−4^
1/16	5.0636 × 10^−2^	9.0239 × 10^−4^	1.1937 × 10^−3^	9.4830 × 10^−4^
1/32	1.8282 × 10^−2^	8.9416 × 10^−4^	9.8137 × 10^−4^	9.4942 × 10^−4^

**Table 4 tab4:** Two-gene system: comparison of average errors for RK3/8, expRK3/8, RK4, and expRK4 methods.

Step size	RK3/8	expRK3/8	RK4	expRK4
1	4.6619 × 10^−2^	8.7331 × 10^−4^	8.6240 × 10^−3^	9.7906 × 10^−4^
1/2	3.7028 × 10^−1^	9.4577 × 10^−4^	5.2468 × 10^−1^	9.4457 × 10^−4^
1/4	3.2666 × 10^−1^	9.4805 × 10^−4^	3.6973 × 10^−1^	9.4766 × 10^−4^
1/8	1.3398 × 10^−1^	9.4894 × 10^−4^	1.4412 × 10^−1^	9.4891 × 10^−4^
1/16	4.4359 × 10^−2^	9.4947 × 10^−4^	4.5572 × 10^−2^	9.4947 × 10^−4^
1/32	1.7338 × 10^−2^	9.4974 × 10^−4^	1.7525 × 10^−2^	9.4974 × 10^−4^

**Table 5 tab5:** p53-mdm2 system: comparison of average errors for Euler, expEuler, Heun, and expHeun methods.

Step size	Euler	expEuler	Heun	expHeun
1/16	3.5455	9.7152 × 10^−3^	3.3784	2.47061 × 10^−3^
1/32	1.3141 × 10^−3^	5.3681 × 10^−3^	1.7446 × 10^−4^	1.2767 × 10^−3^
1/64	6.5825 × 10^−4^	2.7553 × 10^−3^	5.9564 × 10^−5^	4.383 × 10^−4^
1/128	3.0970 × 10^−4^	1.3769 × 10^−3^	4.7031 × 10^−5^	1.1482 × 10^−4^

**Table 6:** p53-mdm2 system: comparison of average errors for RK3/8, expRK3/8, RK4, and expRK4 methods.

Step size	RK3/8	expRK3/8	RK4	expRK4
1/16	3.1651	2.2126 × 10^−4^	3.2096	7.8709 × 10^−4^
1/32	1.2676 × 10^−3^	2.5953 × 10^−5^	1.2770 × 10^−3^	9.4086 × 10^−5^
1/64	6.4492 × 10^−4^	4.2016 × 10^−5^	6.4759 × 10^−4^	4.8393 × 10^−5^
1/128	3.0628 × 10^−4^	4.4244 × 10^−5^	3.0697 × 10^−4^	4.4697 × 10^−5^

## Appendix

### A. Proof of Theorem 4

A fixed point of scheme (12) is a point *y* ^*∗*^ such that *y*_*n*_ = *y* ^*∗*^ implies *y*_*n*+1_ = *y* ^*∗*^. We have assumed that *f*(0) = 0. Suppose that *y*_*n*_ = 0 in scheme (12). The internal stages of scheme (12) define a nonlinear operator Ψ : *Y* ↦ Ψ(*Y*) on *ℝ* ^*sd*^. For *Y*, *Z* ∈ *ℝ* ^*sd*^, the internal stages of system (12) are

(A.1) LY−LZ=h∑j=1sa1jexp⁡c1−cjV·fYj−fZj−ΩYj−Zj,…,∑j=1sasj·exp⁡cs−cjV·fYj−fZj−ΩYj−ZjT≤h∑i,j=1saij·expci−cjV·fYj−fZj+Ω·Yj−Zj≤h∑i,j=1saij·exp⁡ci−cjV·L+ΩYj−Zj≤h∑i,j=1saij·exp⁡ci−cjV·L+Ω·Y−Z=L1Y−Z,

where *L*_1_ = *h*∑_*i*,*j*=1_^*s*^ | *a*_*ij*_ | · ‖exp⁡((*c*_*i*_ − *c*_*j*_)*V*)‖(*L* + ‖*Ω*‖) < 1 if *h* < 1/∑_*i*,*j*=1_^*s*^|*a*_*ij*_| · ‖exp((*c*_*i*_ − *c*_*j*_)*V*)‖ · (*L* + ‖*Ω*‖). Therefore, Ψ is a contraction on *ℝ* ^*sd*^. By the* Banach contraction theorem*, the operator Ψ has a unique fixed point *Y* = 0; that is, *Y*_*i*_ = 0 for *i* = 1,…, *s* which implies that *y*_*n*+1_ = 0. We have proven that the origin *y* ^*∗*^ = 0 is the unique fixed point of the exponential RK method (12).

### B. Analysis of Linear Stability, Distortion, Dispersion, and Dissipation

In this section, in order to examine the behavior of an RK or expRK method Φ_*h*_ : *y*_*n*_ → *y*_*n*+1_ for the dynamical system defined by ODE (3) with a stable steady state *y* ^*∗*^ = 0, we analyze its linear stability and estimate the orders of distortion, dispersion, and dissipation.

Let us consider the linear scalar test equation

(B.1) $$\begin{array}{c} \dot{y}-\Omega y=\varepsilon y, \end{array}$$

where the complex number *Ω* is an estimate of the principal rate and *ε* is the error of the estimation. Applying an RK method (5) or an expRK method (12) to (B.1) yields

(B.2) $$\begin{array}{c} y_{n+1}=R\left(\;V,z\;\right)y_{n},\;V=h\Omega ,\;\;z=h\varepsilon , \end{array}$$

where *R*(*V*, *z*) is called the* stability function* of the method.

For an *s*-stage RK method,

(B.3) $$\begin{array}{c} R\left(\;V,z\;\right)=1+Hb^{T}{\left(\;I_{s}-HA\;\right)}^{-1}e,\;H=V+z, \end{array}$$

where *I*_*s*_ is the *d* × *d* identity matrix and the *s*-dimensional vector *e* = (1,…, 1)^*T*^. For an explicit *s*-stage RK method, since the matrix *A* is an *s* × *s* lower-triangular, then *A* ^*s*^ = 0 and

(B.4) RV,z=1+HbTe−H2bTAe+⋯+−1s−1Hs−1bTAs−1e.

On the other hand, an *s*-stage expRK method (12) has a distortion

(B.5) $$\begin{array}{c} R\left(\;V,z\;\right)=\exp\left(\;V\;\right)+z\exp\left(\;V\;\right)b{\left(\;V\;\right)}^{T}{\left(\;I_{s}-zA\;\right)}^{-1}e. \end{array}$$

If the expRK method is explicit,

(B.6) RV,z=exp⁡V1+zbVTe−z2bVTAe+⋯+−1s−1zsbVTAs−1e.

Definition B.1 B.1. For an *s*-stage method with the stability function *R*(*V*, *z*), the region in the (*V*, *z*) plane

(B.7) $$\begin{array}{c} R_{s}=\left\{\;\left(\;V,z\;\right)\;|\;\left|\;R\left(\;V,z\;\right)\;\right|\leq 1\;\right\} \end{array}$$

is called the* stability region* of the method.

Figure 12 presents the stability regions of RK4 (left) and expRK4, respectively.

Definition B.2 B.2. For an *s*-stage expRK method (5) with the stability function *R*(*V*, *z*), the quantity

(B.8) $$\begin{array}{c} Dist\left(\;H\;\right)=H-\ln R\left(\;V,z\;\right),\;H=V+z \end{array}$$

is called the* distortion* (or* rate error*) of the method. If Dist(*H*) = *𝒪*(*H* ^*k*+1^) as *H* → 0, then the method is called* distortive of order k*. If Dist(*H*) = 0, then the method is called* zero-distortive*.

The distortion of RK4 method is

(B.9) $$\begin{array}{c} Dist\left(\;H\;\right)=\frac{1}{120}H^{5}-\frac{1}{144}H^{6}+O\left(\;H^{7}\;\right),\;H⟶0. \end{array}$$

On the other hand, if we denote the ratio *r* = *ε*/*Ω* = *z*/*V*, then *V* = (1/(1 + *r*))*H* and *z* = (*r*/(1 + *r*))*H* and the distortion of the expRK4 method is

(B.10) DistH=r51201+r5H5−r61441+r6H6+OH7,H⟶0.

It can be seen from (B.9) and (B.10) that RK4 and expRK4 are both distortive of order four. However, if an estimate *ε* of the true rate *Ω* is accurate enough, that is, |*r* | ≪ 1, then the coefficient of the leading term of the distortion Dist(*H*) for the expRK4 method is much smaller than that for the RK method. Moreover, if the frequency can be estimated exactly (*r* = 1), then all the expRK methods are zero-distortive.

It has been observed that most genetic regulatory systems have oscillatory solutions. Therefore it is of importance to measure to what extent a numerical method can preserve the oscillation. In order to compare the accuracy RK methods and that of expRK methods in preserving the oscillatory properties of the exact solution, we assume that *Ω* and *ε* in the test equation (B.1) are purely imaginary; that is, *Ω* = *iλ*, *ε* = *iμ*, *i* ^2^ = −1.

Definition B.3 B.3. For a method with stability function *R*(*V*, *z*), denote the real and imaginary parts of the stability function *R*(*V*, *z*) by *U*(*V*, *z*) and *Q*(*V*, *z*), respectively, where *ξ* = *hλ* and *η* = *hμ*. The quantities

(B.11) Dispθ=θ−arccos⁡Qξ,ηU2ξ,η+Q2ξ,η,Disθ=1−U2ξ,η+Q2ξ,η,θ=ξ+η,

are called the* dispersion* (or* phase lag*) and* dissipation* (or* amplification factor error*) of the method, respectively. If Disp(*θ*) = *𝒪*(*θ* ^*q*+1^) and Dis(*θ*) = *𝒪*(*θ* ^*l*+1^) as *θ* → 0, then the method is called* dispersive of order q* and* dissipative of order l*, respectively. If Disp(*θ*) = 0 and Dis(*θ*) = 0, then the method is called* zero-dispersive* and* zero-dissipative*, respectively.

For both RK4 and RK3/8, the dispersion and dissipation satisfy

(B.12) Dispθ=−θ5120+Oθ7,Disθ=θ6144+Oθ8.

Therefore, RK4 and RK3/8 both are dispersive of order four and dissipative of order five.

Let *r* = *μ*/*λ*. Then the dispersion and dissipation for both expRK4 and expRK3/8 are

(B.13) Dispθ=−r51+3r+r21201+r6θ6+Oθ8,Disθ=r61441+r6θ6+Oθ8,θ⟶0.

Therefore, expRK4 and expRK3/8 both are dispersive of order five and dissipative of order five. Then expRK4 (expRK3/8) has dispersion of one order higher than RK4 (RK3/8). If |*r* | ≪ 1, then the coefficient of the leading term of the dissipation Diss(*θ*) for the expRK4 (expRK3/8) method is much smaller than that for RK4 (RK3/8). Furthermore, if *r* = 1, then all the expRK methods are zero-dispersive and zero-dissipative.

The above analysis explains why, when the rate *Ω* of the problem is estimated accurately enough, an expRK method is more accurate than its prototype RK method in preserving the distortion, dispersion, and dissipation of the exact solution.
